# Recurrent Malignant Sweat Gland Tumor

**DOI:** 10.7759/cureus.55047

**Published:** 2024-02-27

**Authors:** Kavya A, Mahendra Wante, Dakshayani S Nirhale

**Affiliations:** 1 General Surgery, Dr. D. Y. Patil Medical College, Hospital & Research Centre, Pune, IND

**Keywords:** rare skin tumor, recurrent swelling, hidradenoma, hidradenocarcinoma, adnexal tumor

## Abstract

Malignant sweat gland tumors are very rare. Hidradenocarcinoma is an uncommon malignancy arising from the intradermal ductal epithelium of eccrine sweat glands, usually in the sun-exposed parts of the body. It usually arises de novo but may develop from a benign hidradenoma. The diagnosis of hidradenocarcinoma is clinically challenging as it presents with varied consistency and clinically mimics other skin lesions such as chronic sebaceous cysts or epidermoid cysts. Hidradenocarcinoma is a highly aggressive tumor with a tendency for regional and distant spread. It is difficult to treat hidradenocarcinoma as it has high rates of morbidity and mortality and a very high incidence of recurrence. Here, we report a rare case of a 45-year-old woman who presented with a recurrent lump over the left arm diagnosed as primary hidradenocarcinoma.

## Introduction

Skin adnexal tumors encompass a wide variety of benign and malignant disorders, which are classified according to their differentiation, reflecting their origin from hair follicles, sweat glands, or sebaceous glands. They serve as indicators for several syndromes, including Murie-Torre and Cowden syndromes. Hidradenocarcinoma is an uncommon type of malignant adnexal tumor that develops from eccrine sweat glands. It represents 0.001% of all tumors and 6% of all malignant eccrine tumors [1]. Based on histology, it is frequently referred to as clear-cell eccrine carcinoma, primary mucoepidermoid cutaneous cancer, malignant clear-cell acrospiroma, or malignant clear-cell hidradenoma [2]. Although sometimes a benign hidradenoma undergoes malignant transformation, hidradenocarcinoma often develops sporadically. Areas of the head and neck, particularly the face, receiving significant sun exposure are most commonly affected. Hidradenocarcinoma in the limbs is an uncommon occurrence [3]. The majority of cases occur in females and often in the fifth to seventh decade of life [4]. No identified risk factors exist. It often manifests as a cutaneous tumor with solid, cystic, nodular, or variable consistency. It grows slowly and is occasionally accompanied by skin abnormalities, including erythema, ulceration, or drainage [5]. It is an aggressive tumor with a significant risk of local recurrence and distant spread. With just about a 30% five-year survival rate, it has a dismal prognosis [6].

## Case presentation

**Figure 1:**
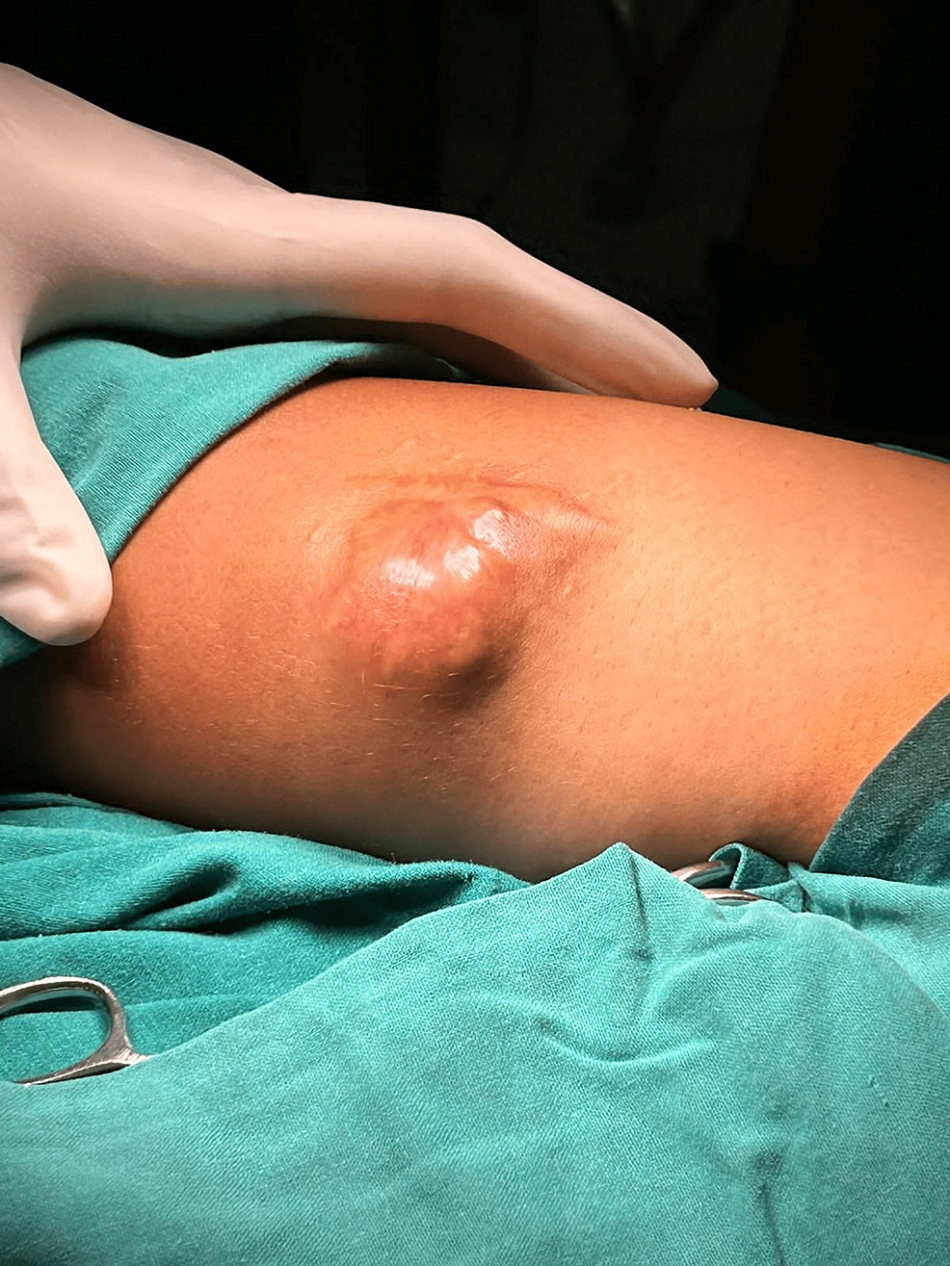
Clinical presentation of hidradenocarcinoma over the posterolateral aspect of the left arm.

A 45-year-old female presented to the surgical outpatient department with a solitary swelling over the lateral aspect of the left arm for one year. The patient provided a history of similar swelling at the same site twice before for which she underwent excision. However, histopathological reports were not available. On examination, a 4 × 5 cm swelling was noted on the posterolateral aspect of the left upper arm which was mobile, non-tender, and had variable consistency (Figure 1). Left axillary lymph nodes were not palpable.

Ultrasonography of local swelling revealed a well-defined oval encapsulated heterogeneously hypoechoic cystic lesion measuring 40 × 37 × 45 mm (craniocaudal × anteroposterior × transverse) with thick septae and internal echoes noted in the subcutaneous plane in the posterolateral aspect of the left arm with minimal peripheral vascularity on color Doppler, suggestive of an epidermal cyst with infective changes. Fine-needle aspiration cytology was suggestive of a chronic sebaceous cyst.

**Figure 2:**
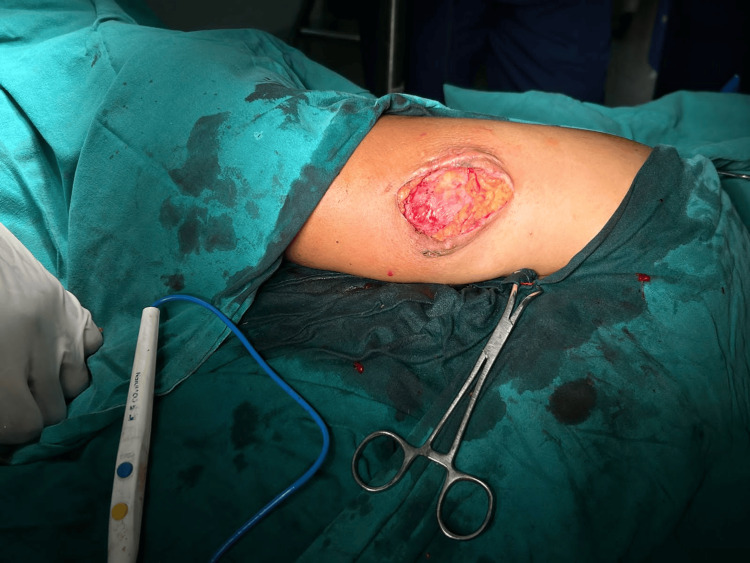
Wide local excision of swelling over the left arm.

**Figure 3:**
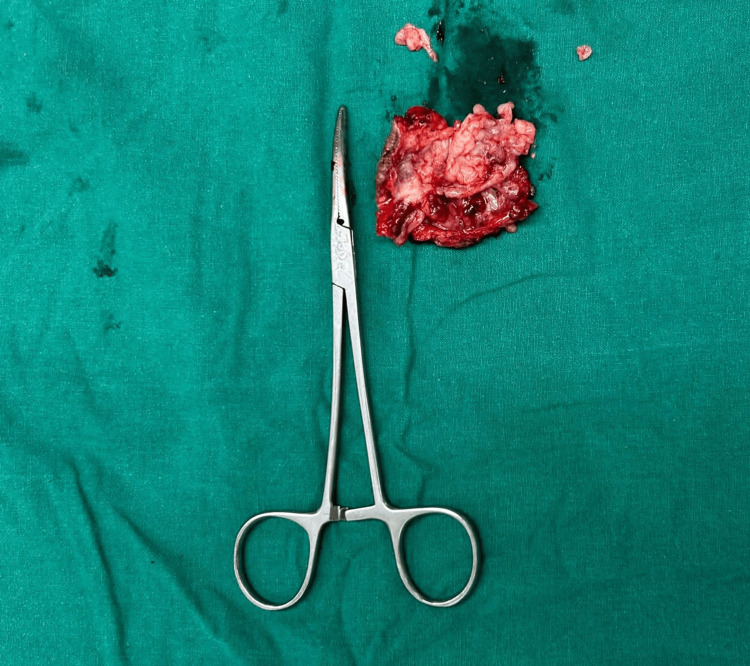
Excised specimen of the lump which was sent for histopathological examination.

**Figure 4:**
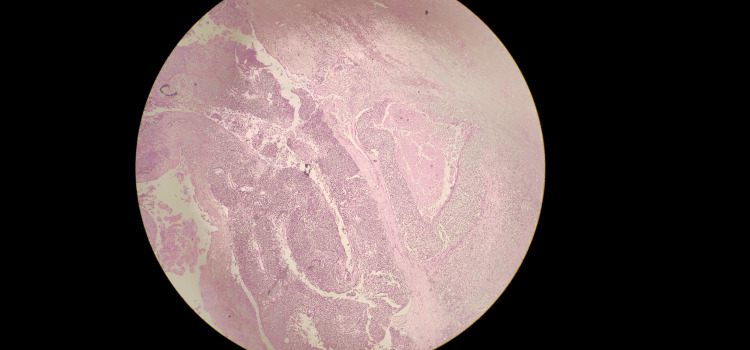
Histopathology of the excised tumor showing a fairly circumscribed tumor composed of cells arranged in nodules and sheets.

**Figure 5:**
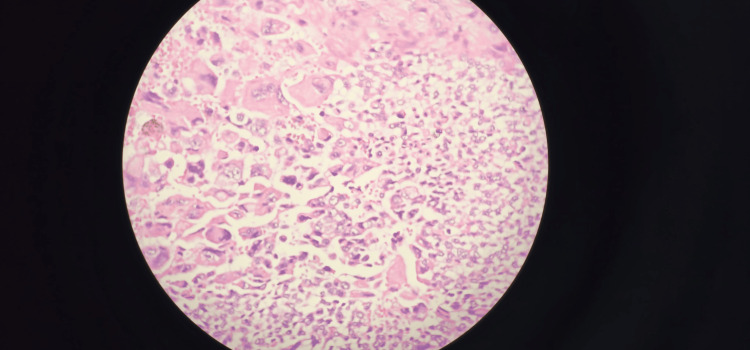
Histopathology of the excised tumor showing polygonal cells with a moderate amount of clear cytoplasm, central round-to-oval vesicular nuclei with few showing distinct nucleoli. Frequent mitoses with atypical mitoses can be seen. Foci of necrosis can be seen. Focal ductal differentiation can be seen. No evidence of lymphovascular emboli or perineural invasion.

Considering recurrence and variegated consistency, the decision was made to perform a wide local excision of the swelling. A 1 cm margin on all sides was given clearance (Figures 2, 3). A frozen section of the excised specimen revealed a skin adnexal tumor with tumor-free margins. The final histopathological examination revealed hidradenocarcinoma with free margins and the absence of perineural and lymphovascular invasion (Figures 4, 5). The postoperative period was uneventful.

**Figure 6:**
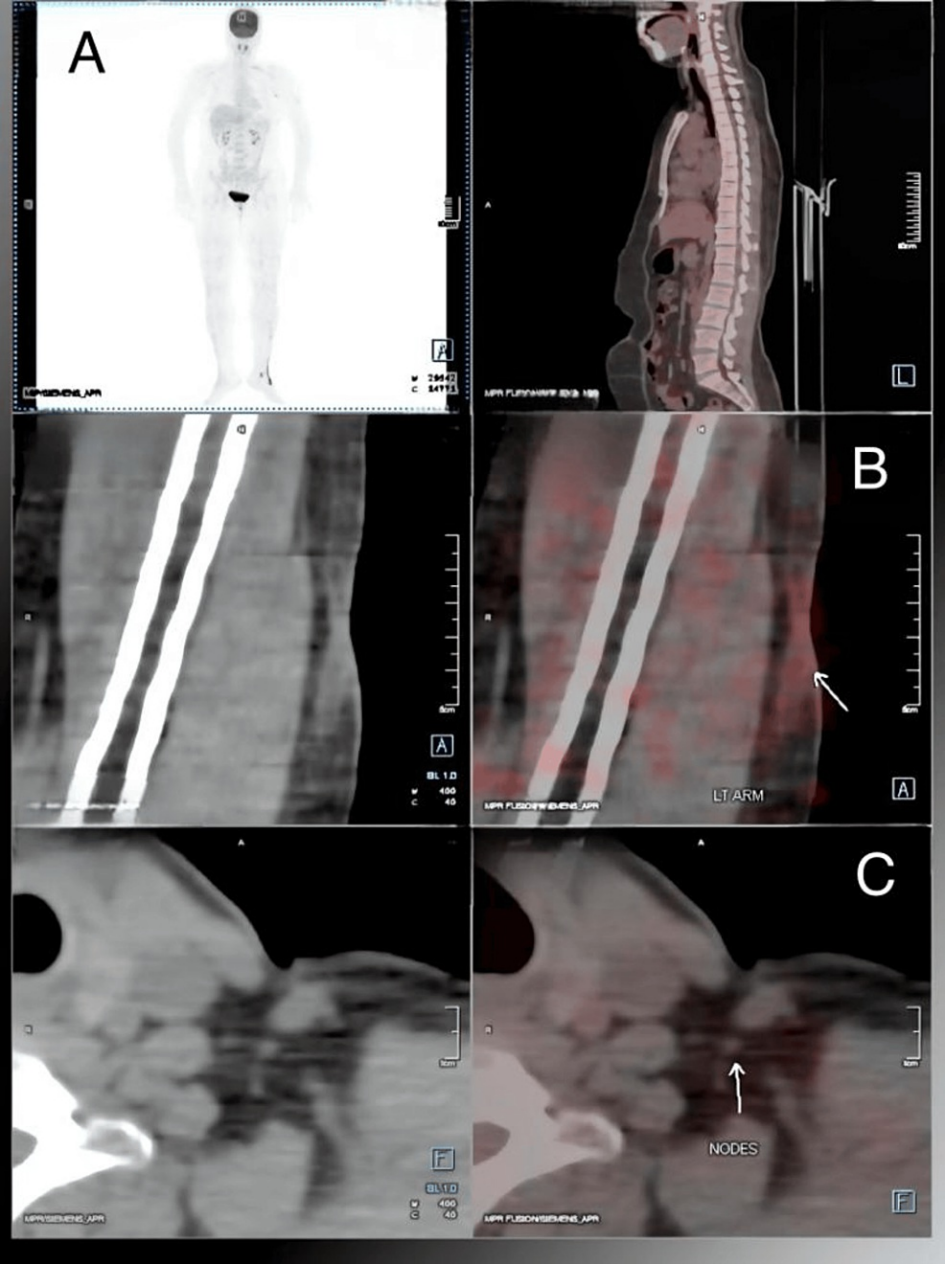
Positron emission tomography-computed tomography scan. (A) Physiological uptake of the tracer. (B) Weakly metabolic cutaneous-subcutaneous thickening in the outer aspect of the left arm. (C) Ametabolic left axillary nodes.

A positron emission tomography-computed tomography scan done at the one-month follow-up revealed weakly metabolic cutaneous-subcutaneous thickening in the outer aspect of the left arm with ametabolic left axillary nodes. No 18-fluorodeoxyglucose-avid distant organ involvement was noted (Figure 6). The patient continues to be in close follow-up.

## Discussion

Hidradenocarcinoma is an uncommon type of malignant soft tissue tumor that develops from eccrine sweat glands. More commonly, hidradenocarcinoma develops sporadically, but sometimes a benign hidradenoma can also undergo malignant transformation. Clinically, it manifests in a variety of forms that are not very different from other lesions. Hidradenocarcinoma usually presents as a solitary, firm nodule that is neither mobile nor tender. On the other hand, they can occasionally be cystic or multilobular and are linked to other skin abnormalities. The differential diagnosis frequently includes chronic sebaceous cysts, epidermoid cysts, melanoma, fundibular cysts, basal cell carcinoma, dermatofibroma, squamous cell carcinoma, dermatofibrosarcoma protuberans, pyogenic granulomas, pilar cysts, and glomus tumor. They usually occur on sun-exposed parts such as the head and neck region and are seldom found on the extremities. It is a highly aggressive tumor, with a reported nodal involvement rate of 39% and metastatic rates ranging from 28% to 60%. Currently, wide local excision of the lesion is the most common and the initial treatment for hidradenocarcinoma. However, surgical excision alone has been shown to have a 10% to 50% recurrence rate. High rates of metastasis necessitate surgical excision of involved lymph nodes for optimal treatment [7]. Radiation and chemotherapy should be considered in inoperable cases, or in cases that have positive margins, recurring tumors, and lymph node metastases. The postoperative five-year survival rate has been less than 30%, according to Souvatzidis et al. [8]. For a better prognosis, early detection, diagnosis, and surgical intervention are crucial.

## Conclusions

Hidradenocarcinoma is a rare and aggressive sweat gland tumor with high rates of local recurrence and distant metastasis. Hidradenocarcinoma poses a significant diagnostic challenge due to its clinical mimicry of other benign lesions such as chronic sebaceous cysts or epidermoid cysts leading to misdiagnosis and mistreatment, potentially worsening the prognosis. This emphasizes the importance of considering hidradenocarcinoma in the differential diagnosis for recurrent swellings and advocating for wide local excision as the primary treatment modality. Histopathology remains the gold standard for definitive diagnosis. Additional treatment modalities, such as radiation therapy or chemotherapy, may be recommended depending on the individual case.
